# Fully Inkjet-Printed CuO Sensor on Flexible Polymer Substrate for Alcohol Vapours and Humidity Sensing at Room Temperature

**DOI:** 10.3390/s19143068

**Published:** 2019-07-11

**Authors:** Petr Krcmar, Ivo Kuritka, Jan Maslik, Pavel Urbanek, Pavel Bazant, Michal Machovsky, Pavol Suly, Petr Merka

**Affiliations:** Centre of Polymer Systems, University Institute, Tomas Bata University in Zlin, trida Tomase Bati 5678, 760 01 Zlin, Czech Republic

**Keywords:** copper oxide, nanoparticles, inkjet, gas sensor, polymer substrate, low temperature, sensing mechanism

## Abstract

This work focuses on an inkjet-fabricated sensor based on copper oxide nanostructured particles on polymer flexible substrate for the sensing of alcohol vapours and humidity at room temperature. Nanoparticles were prepared by a microwave-assisted solvothermal sealed vessel synthesis method. The ink composition was developed on the basis of viscosity and surface tension optimization by the addition of polymeric steric surfactant and dispersant. The printing process was optimized with the help of non-dimensional criteria. Silver nanoink was used for the printing of an interdigitated pattern on a PET substrate which was overprinted by the copper oxide ink, thus obtaining a flexible flat sensor. Material design and all fabrication steps of the sensor respected the temperature limitation given by the thermal stability of the polymer substrate. Printed layers and motifs were characterized microscopically and by resistance measurement. The effectiveness of the prepared sensor was demonstrated and studied by measuring the response to saturated vapours at room temperature. The sensing layer showed the opposite resistance response to stimuli than expected for the well-known p-type sensing mechanism of CuO sensors operated at high temperatures. In addition to vapour sorption, condensation and desorption influencing electron, proton and ionic conductivity, manifestation of another mechanism was observed and an explanation suggested in terms of the electrochemical mechanism.

## 1. Introduction

Inkjet printing is a well-known technology which has been originally focused to transfer electronic graphical data onto substrates, like paper and flexible polymer sheets. Inkjet printing is an additive manufacturing non-contact technique which enables transfer of graphical data to a target substrate by digitally-controlled deposition of droplets of ink. Therefore, this method can be used not only for graphics or text printing but it can be used more generally to deposit materials onto any flat surface. The material can be deposited according to the desired digital motif or pattern, which is called material printing. This technology has already been successfully used for the production of various electronic components, sensors, optical component of flexible displays and OLED devices [1,2,3]. A plethora of inorganic solid compounds can be deposited by this non-contact digital technique if they are prepared in the form of a fluid nanosuspension. Among such nanomaterials, copper(II) oxide (CuO) is an important p-type semiconductor metal oxide. Among the studies of transition metal oxides (MOs) CuO has become a hot topic due to its interesting properties. It is a p-type semiconductor material with a relatively narrow band gap of ~1.2 eV (in bulk). Nanostructures based on CuO possessing large specific surface areas and manifesting dimension-scale effects were studied because of their excellent physical and chemical properties that are remarkably different from those of their micro-scale or bulk volume analogues. Such nanostructures are also considered as electrode materials suitable for batteries and as promising materials for application in solar cells due to their high absorbance in the solar radiation spectral range, low thermal emissivity and relatively high charge carrier mobility and concentration. Moreover, CuO-based nanostructures are a subject of extensive study for various other applications, among them bio-sensors, nanofluids, photodetectors, energetic materials, field emitters, supercapacitors, photocatalysis and removal of pollutants, magnetic storage media and gas and humidity sensors [4,5,6,7]. Hierarchical nanostructures with p-n junctions of CuO and ZnO can be prepared, as well [8].

Humidity sensors are an important class of devices that have been used extensively in practice. For example, these sensors can be used for monitoring of the environmental (outdoor) or interior (indoor) moisture in buildings, cars, medicine, construction and engineering, semiconductor fabrication, food processing and related industries, as well as in meteorology. In addition to dry-wet thermometers, other conventional methods are available to measure relative humidity. They are based on monitoring the changes in the oscillation frequency of piezoelectric elements or variations of the luminescence intensity of micro-porous thin films in response to changes in the relative humidity. Relative humidity can be also determined by measuring the resistance or the capacitance of thin moisture-sensitive films made from materials such as polymers and ceramics [9,10,11,12,13,14,15,16].

Similarly to the moisture-sensitive elements, sensors for volatile organic compounds (VOC) can be fabricated with the use of CuO material. The gas-sensing properties of such sensors were investigated. Vapours of ethanol, acetone or benzene were examined as representative VOCs. Methods of sensing device preparation have been successfully demonstrated [17]. Among them, prepared sensors can be used in analytical applications for the monitoring of gas or air composition or pollution, and environmental safety. Additionally, such sensors may be applied in process control in biotechnology like ethanol or acetic acid production and for product control, during transport and shelf life of goods, namely food (ripening of fruits, etc.).

A principal limitation in the fabrication and operation of flexible sensors printed on polymer substrate is the upper temperature limit. Normally, even very good polymer films cannot survive temperatures higher than 300 °C or 400 °C for high-performance materials, like polyimides, without being significantly degraded. Thus, high-temperature annealing and sintering fabrication steps at temperatures common for sensors fabricated on inorganic substrates are not applicable to polymer-based devices. Operation temperatures of about 100 °C can be standardly accessible for polymer-based devices [18]. Nevertheless, a fully-printed sensor on a plastic substrate working up to at least 300 °C has been already demonstrated [19]. On the other hand, a strong motivation for a low-temperature operation mode requirement might be the character of the application itself, especially if the device is in direct contact with living organisms (body temperature) or for low power consumption applications at room temperature, as successfully demonstrated for CuO/graphene material [20]. While there is a concise picture of the sensing mechanism of CuO humidity- and VOC-based sensors operating at higher temperature [6], only rare reports are available on their behaviour at ambient temperature which seriously deviates from standard expectations [21,22]. To summarize, formulation of suitable inks, smart engineering of the fabrication process and understanding the low-temperature sensing mechanism represent the greatest challenges in this field nowadays.

In this paper, we demonstrate the preparation of a fully inkjet-printed gas humidity and VOC (alcohols) CuO-based sensor on a common flexible poly (ethylene terephthalate) (PET) substrate. In addition to the practical aspects of the development of a new sensor, including microwave-assisted synthesis of the material, ink formulation and printing, it is worth studying its sensing mechanism at low temperature. We show that CuO imparts a more complicated sensing mechanism at room temperature, different from the common p-type semiconductor mechanism applied in high-temperature operating CuO-based sensors. Moreover, unlike commercial low-cost humidity sensors based on ceramics or alumina, the bare polymer substrate has no response to moisture or VOCs and the sensing mechanism is not only based on the condensation in the porous structure of the printed sensing layer. Another important issue which is to be addressed is the condition that the prepared device cannot be fired at high temperatures to achieve sintering and remove volatilize-able components of the ink due to the limited temperature stability of the polymeric substrate. Additives or auxiliary components that cannot be removed by annealing at relatively low temperatures, even with the help of vacuum, residues in the prepared layers and possibly affect the sensing mechanism as well. Finally, the method of sensing response reading can influence the sensing mechanism, too, since the device is part of the electric circuit and powered to measure its resistance.

## 2. Materials and Methods

### 2.1. Synthesis and Characterization of CuO Nanoparticles

CuO nanoparticles were prepared via the simple water-ethyleneglycol (water-EG) solvothermal microwave-assisted method. The method was adopted from [23] and improved by utilization of microwaves [24]. Cupric chloride dihydrate (CuCl_2_∙2H_2_O) was used as a precursor. Potassium hydroxide (KOH) was used as a precipitating agent. Neither surfactants nor any template were used. All chemicals were of analytical purity and used as received from Sigma-Aldrich s.r.o. (Prague, Czech Republic) Typical experimental procedure was carried out this way: CuCl_2_∙2H_2_O (1 g) was dissolved in distilled water (54 mL). Then, an appropriate amount of EG (6 mL) and KOH (3 g) was added in turns. The as-prepared solution was poured into the Teflon vessel proper to use in a microwave oven (MARS 5, CEM Corporation, Matthews, NC, USA). Vessels with solution were sealed to ensure appropriate pressure inside. The reaction was maintained at 100 °C under 110 kPa for 30 min. After reaction, the product was washed by distilled water and absolute ethanol several times in order to remove eventual impurities. The prepared powder was dried for 20 h at 60 °C.

Characterization of the prepared powder was performed by a Nova NanoSEM 450 scanning electron microscope and by powder X-ray diffraction (XRD) with the aid of an X’Pert PRO X-ray diffractometer (PANalytical, Almelo, The Netherlands) using Cu Kα radiation of *λ* = 0.15406 nm.

### 2.2. Ink Formulation and Characterization

The series of inkjet inks was prepared from copper(II) oxide nanoparticles by dispersing them in a mixture of a surfactant and a dispersant in various ratios and a constant volume of water. Both surfactant and dispersant BYK^®^ 348 and DISPERBYK^®^ 190 were supplied by BYK Additives and Instruments, a member of ALTANA, Wesel, Germany. The surfactant BYK^®^ 348 is a polyether-modified siloxane with non-volatile matter content higher than 96 wt% as declared by the supplier. The dispersant DISPERBYK^®^ 190 is a water solution of a high molecular weight block co-polymer with pigment affine groups and its non-volatile matter content is 40 wt% (as declared by the supplier estimated by drying for 10 min at 150 °C). The dispersant has acid value 10 mg KOH/g as declared by the supplier while no amines are present. The dispersion process was agitated by an ultrasound apparatus working at a 50:50 duty cycle with the full power and five minutes (GM 20170, Bandelin Electronic, Sonopuls Rosette cell) The concentration of CuO varied in the range of 3–10 wt% and the amount of polymeric dispersant and surfactant was set in the range 5–35 wt%. Five ink compositions were prepared. The viscosity, density and surface tension were measured at 25 °C. The surface tension was determined using a Krüss K100force tensiometer. The Wilhelmy plate method was used for measurements in all cases of surface tension of examined solutions. The viscosity of dispersions was measured by Lovis 2000 M/ME microviscometer at 25 °C. The diameter of the used capillary tube was 1.8 mm and 1.5 mm for the gold ball.

### 2.3. Printing of Thin Films, Patterns and Sensor Specimens

**Figure 1:**
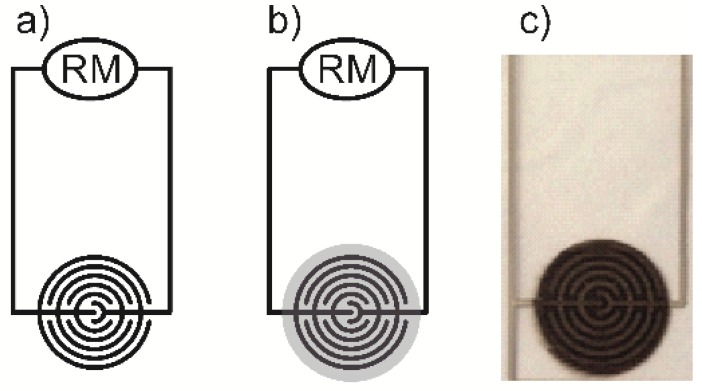
A photograph and s scheme of the sensor and its connection in the circuit. RM—resistance meter, (**a**) without the sensitive CuO layer, (**b**) with the sensitive CuO layer and (**c**) a real photograph of the sensor.

Aqueous dispersion of Ag nanoparticles (Metalon JS-B25HV, 25 wt%) for electrodes was delivered by Novacentrix, US. As substrates, PET foils (product name: NoveleTM IJ-220) were used for material printing and supplied by Novacentrix, US, as well. All obtained dispersions were tested for deposition by FUJIFILM DMP-2800 series Dimatix Materials Printer. A single square of 2 × 2 cm was used as the testing motif printed on the PET substrate to evaluate preliminary the conductivity (by four-point probe) of films printed from tested inks and silver dispersion. Ink 4 was found to be the best performing for printing and the most stable dispersion. Therefore, it was used for fabrication of the sensor devices intended for further testing. The device was fabricated by the same material printer. The printing motif is depicted in Figure 1. An interdigitated silver electrode pattern from the commercially available Metalon dispersion was printed on the flexible PET substrate. Printed electrode patterns were dried in a vacuum oven at 80 °C for 20 min. To achieve good quality of the electrodes, the first layer was trapped by another one under the same conditions. After that, continuous thin CuO film discs were printed on the top of this patterned substrate to form the set of sensing devices. A sheet with printed devices was dried once again under the same conditions as the electrodes. This process was repeated five times to obtain films of sufficient coverage, thickness and quality. Finally, single devices were cut from the printout sheet.

### 2.4. Analysis of Printed Patterns

The surfaces of printed patterns were analysed by optical microscope, a LEICA DVM25000 Digital Camera, Dimension ICON atomic force microscope in ScanAsyst mode, a Nova NanoSEM 450 scanning electron microscope and a Bruker ContourGT optical profilometer.

### 2.5. Electrical and Sensing Properties

Measurements of printed film conductivity were performed by the use of van der Pauw four-point probe method. The apparatus comprised a Keithley ammeter 6517B, Keithley source 2410, and Keithley switch 7002. The probe contacts were coated with gold. A square geometry of the samples with contacts joined at the corners was used.

The electrical resistance of the sensors was measured with the aid of a UNI-T HC-UT71D multimeter. Figure 1 shows a scheme of the prepared gas sensor connected to the resistance meter. The holder with the sensor specimen was swiftly transferred into a flask with saturated vapours of alcohol or water. After 5 min of adsorption, the signal was stabilised (on-state) and the sensor specimen was then quickly removed from the flask and the measurement of the sample was continued in the desorption mode of on the ambient air atmosphere until stabilised (off-state) which took another 5 min. The sensor response is calculated as the relative change (in percent, %) of the resistance of the sensor (Δ*R/R*_0_). The initial value (at time zero) was difficult to measure for the sensor freshly removed from the storage conditions (stored in a cabinet-like desiccator with RH in the range of 20–30%) since it is the first measurable value below the upper measurement range of the multimeter, which is 40 MΩ. Therefore, 40 MΩ was chosen as the *R*_0_ value in order to allow for a comparison of sensor responses. Humidity in the laboratory ambient atmosphere was not under control and can be considered as an eventual source of baseline drift in records of sensor responses for alcoholic vapours. In the case of humidity sensing, the sensor actually responds to the change between ambient humidity (measured by a laboratory hygrometer, 45% RH) and 100% RH in saturated vapours. In the case of alcohol sensing, the ambient concentration of vapours was zero while it was 100% in saturated vapours, and the ambient humidity was about 30% RH. The resistance measurement of each sensor was always performed for three such adsorption–desorption cycles. All measurements were conducted at ambient atmospheric pressure and a laboratory temperature of 25 °C.

Recording of I-V characteristics was performed with the aid of a home-built setup of an HP 34401A multimeter (Hewlett Packard, Palo Alto, CA, USA) utilized for current measurement and n HP 6038A power supply (Hewlett Packard, Palo Alto, CA, USA) operated in the regime of voltage control. In this case, the specimens were not stored in a desiccator, but stabilised at the ambient atmosphere in yet another laboratory. The humidity there was about 70% RH at the time of the measurements. Both apparatus were controlled by a PC using the Labview application.

## 3. Results and Discussions

Since the work represents a joint study on the device fabrication process and properties of prepared sensors, this section is divided into two parts. Firstly, experimental results gathered during the ink formulation, printing process development and device fabrication are described and discussed. Next, the results of the characterization of the electrical and sensing properties of the prepared devices are interpreted with emphasis put on the sensing mechanism.

### 3.1. Results and Discussion of Material and Process Development and Device Fabrication Process

#### 3.1.1. Characterization of CuO Particles

**Figure 2:**
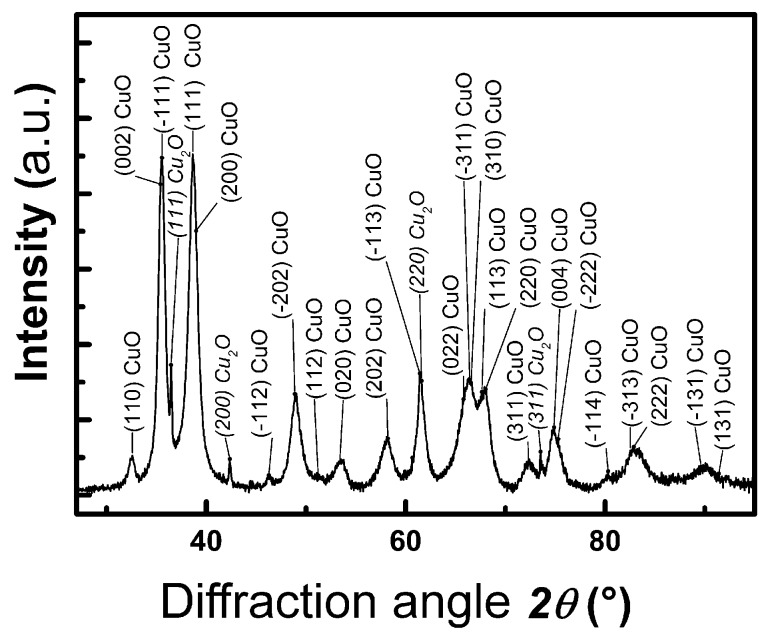
XRD analysis of prepared powder material.

The results obtained by XRD analysis of prepared powder material are presented in Figure 2. Copper oxide CuO monoclinic phase (*C*2/*c*) was confirmed (according to JCPDS no. 01-080-0076) as the prevailing crystalline phase in the material. There are only four peaks of very low intensity in the diffractogram, which can be attributed (according to JCPDS no. 01-071-4310) to the Cu_2_O cubic phase (Pn-3m) thus manifesting in the presence of the trace concentration of this phase in the prepared material. The evident broadness of the recorded diffraction lines testifies to the nanosize of diffracting crystallites although no further analysis of the data was performed for this sake.

**Figure 3:**
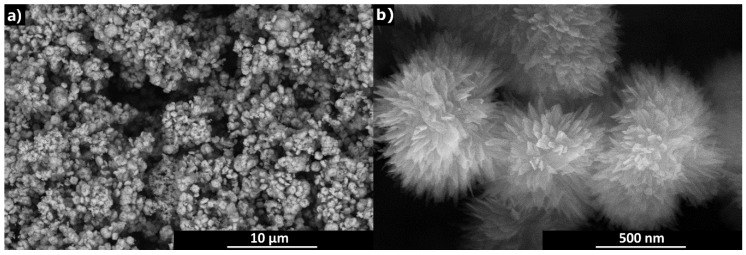
SEM images of CuO nanoparticles at low (**a**) and high (**b**) magnification.

In Figure 3, it can be seen that copper oxide nanoparticles have characteristic morphology of chrysanthemum flowers with large surface area which gives the best chance of the material to be sensitive to any gaseous substance. Superiority of the nanostructured CuO sensors was demonstrated for nanowires [25] and nanostructured films [26] both experimentally and theoretically. The size of individual petals is about several hundred nanometres in length, the width of petals ranges from several tens to one hundred nanometres and their thickness can be roughly estimated at less than a few tens of nanometres.

#### 3.1.2. Ink Formulation and Characterization

Firstly, it is important to note that prepared inks did not meet the required surface tension in the range of 28–42 mNm^−1^, viscosity in the range of 10–12 mPa∙s and particle size below 0.2 µm as suggested by the material printer Dimatix DMP 2800 series producer for optimal use of inks in ink jet printing using an original 10 pL printing head. Each printing nozzle orificium on the head has a square shape with the side size of about 21.5 μm. However, according to the producer, the viable viscosity range may be extended. Thus, it is possible to print a large variety of fluids in the range of the viscosity value from about 1 mPa∙s for water-like fluids up to 30 mPa∙s typical for low viscosity oils. Similarly, the surface tension range may be extended from 20 mNm^−1^ up to about 70 mNm^−1^.

**Figure 4:**
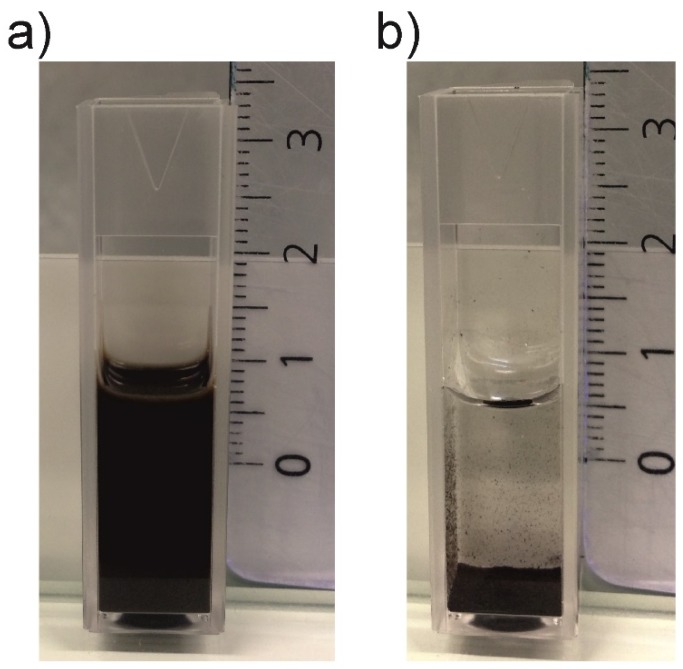
Ink stability (**a**) aqueous dispersion of CuO with additives after three weeks; and (**b**) aqueous dispersion of CuO without additives after one hour.

Table 1 shows the experimental values of the surface tension and viscosity of prepared CuO inks. The dispersions were prepared at various concentrations of the surfactant and dispersant. As can be seen, additives substantially affect the surface tension in the desired way. The addition of surfactant significantly decreases the surface tension of aqueous systems. In this way, the surfactant improves particularly wetting of the substrate and levelling. On the other hand, dispersant additives deflocculates particles through the steric stabilization of the particles. Both of these components reduce viscosity, thus the levelling is also improved and higher particle loading is possible. Figure 4 illustrates the ink stability exemplified on aqueous dispersions of CuO with and without additives. It is evident that the ink with additives is stable over weeks while the dispersion without additives settled within several tens of minutes. The surface tension of the ink plays an important role in the formation of droplets and adhesion to the substrate. The surface tension did not significantly vary between different inks and was around 22 mNm^−1^, which is in the processing range of 20–40 × 10^−3^ N∙m^−1^ recommended by the printer producer.

#### 3.1.3. Refinement of the Ink and Inkjet Process Development by Dimensionless Criteria Analysis

In addition to material properties, process and tool parameters must be taken into consideration in order to develop the ink formulation and printing process rationally. The primary process parameter is the fluid ejection velocity can be approached by the droplets velocity as observed by the drop watch camera integrated in the printer. The main tool related parameter is the printing nozzle orificium equivalent diameter (which is the characteristic length). Its value 21.5 μm is fixed in this case. A framework of dimensionless criteria analysis in the Oh vs. Re space was used similarly as in our previous work [27]. Table 2 summarizes the values of dimensionless criteria calculated for the prepared ink dispersions under conditions of the real printing process, i.e., a fluid velocity of 4 m/s.

**Figure 5:**
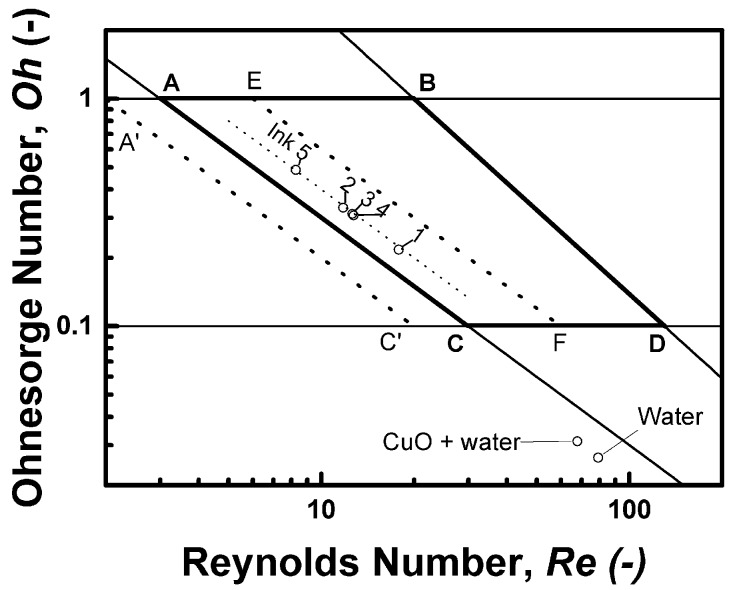
Printability window and positions of prepared inks in the *Oh* vs. *Re* space, for detailed description see text.

Figure 5 shows the positions of prepared inks in the *Oh* vs. *Re* space. The quadrangle ABCD defines a region in which the particular fluids are printable and single drop formation can be achieved or merging with the satellite can be expected, according to McKinley and Renardy [28] who redrew the schematic diagram originally constructed by Derby [29]. The line AC corresponds exactly to the value of *We* = 9 (actually *We*^1/2^ = 3). Derby himself again raised the importance of the *We* criterion in [30] where he published a corrected version of the graph for printability assessment. Our experience is in accord with his achievements and stressed out in our previous work too. Thus, the *We* value of 4 used instead of 9 as a minimum Weber number for a drop generation corresponds to the dotted line A’C’ with the slope of −1 corresponding to *We*^1/2^ = 2 in the graph [27]. Tested inks are represented by five empty circle data points labelled in accordance with Table 2. Points for water and for the CuO dispersion in water are displayed, too. Note that the experimental points are aligned in a virtual line (marked by a dot line in the graph). This line has the slope of −1, which represents a constant value of *We*^1/2^ = 4. In order to assess the effect of the fluid velocity, a dotted line EF with the slope −1 corresponding to *We*^1/2^ = 6 is shown for our inks as if they have a drop velocity of 6 m/s.

A group of inks (Inks 2–4) can be found in the centre of the graph in Figure 5. Nevertheless, Ink 4 performed better than Inks 3 and 2 under otherwise nearly the same conditions, which points towards the importance of the internal material (structural) parameters of the ink. Since the Weber number evaluates surface tension and inertia effects, and does not employ viscosity, the use of the capillary number (*Ca*) can be considered. Although the *Ca* includes viscosity, which is an internal structural parameter, it does not help to resolve between Inks 2–4 (see Table 2). Most likely, the ink fluid is viscoelastic to a certain extent, and the elasticity contributes to the drop formation together with the surface tension; however, deeper analysis in that manner is beyond the scope of this work and remains a challenge for eventual further research.

#### 3.1.4. Printing Process with a Dimatix DMP 2800 Series Device

The piezoelectric printing process is individually controlled for each nozzle by a pulse waveform that consists of several stages that are defined by the slew rate, duration, and magnitude of the pulse voltage. A suitable setting of the waveform leads to good droplet generation, printing rate and uniformity of printed layers. The particular segments were carefully adjusted so that a uniform droplet rate was achieved. Figure S1 (in the Supplementary Information) shows the droplet formation and the waveform developed for CuO ink. The velocity of droplets was about 4 m·s^−1^ at defined conditions (which is significantly less than 6 m·s^−1^ advised by the printer producer for typical printing). As can be seen, jetting of single droplets is uniform with tails but without satellites. The tail is present only at the start of jetting and it blends into the droplet afterwards, hence the precision of printing is assured. The cartridge temperature 30 °C, a substrate temperature 55–60 °C, and voltage 24 V approximately and individually fine-tuned for each nozzle led to optimal jetting conditions. The resolution was adjusted to 1280 dpi, which corresponds to drop spacing in a size of the half dot diameter. The printing of interdigitated electrodes by nanosilver ink was controlled by a suitable waveform and other setup conditions as well, however, this is of no merit to the topic of the article and is skipped for the sake of brevity.

#### 3.1.5. Analysis of the Printed Patterns

**Figure 6:**
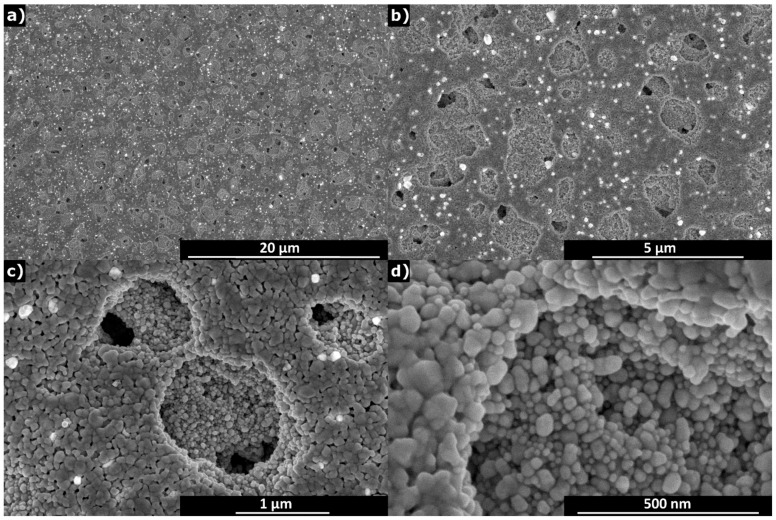
SEM images of printed Ag layers at different magnifications increasing from (**a**) to (**d**).

The structure of the printed Ag layers was analysed microscopically. Figure S2 in the Supplementary Information shows part of the interdigit surface captured by optical microscope. The results of SEM observations are shown in Figure 6 in different magnifications. The dimensions of silver particles are below 100 nm. The printed layer was dried at 80 °C in vacuum in order to achieve better homogeneity of layers and impart good conductivity to electrodes. It can be seen that there are some micropores in size about 1 μm but they are sparsely distributed that it cannot limit the conductivity of the printed lines of much larger dimensions. As seen in the image with the highest magnification, silver particles are densely packed and congruent enough to assure contiguity of the printed material pattern.

**Figure 7:**
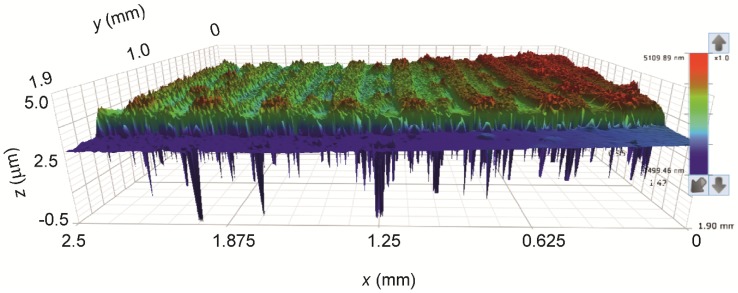
3D topography of printed Ag layers.

Tens and hundreds of micrometres are the proper scale for study of the device’s morphology. The 3D topography of printed Ag layers is shown in Figure 7. A relatively high roughness of the surface is evident; however, it must be taken into account that negative peaks in the graph are artefacts caused by porosity of the printed layers. Additionally, however, two layers of silver are enough to assure sufficient electrical properties of the printed electrode motif and, therefore, any other overlay prints or post treatment procedures were not applied. The overall ridge morphology of the silver electrode surface is a result of chosen drop spacing and dot size, which causes overlay and coalescence of printed droplets. Since the printing head has 16 nozzles and the resolution was set at 1280 dpi, this means that one printed row is 0.3175 mm wide, theoretically. The trapping of layers was intentionally shifted by approximately one half of the row width to assure efficient overlay of the material in both directions throughout the printed motif. Indeed, the analysis of the printed motif profiles shown in Figure S3 in the Supplemental Information confirmed the periodicity of lines to be 0.32 mm and the shift between the lower and upper layers is found to be 40% of the line width. The upper graph panel in the supplementary figure confirms good continuity of the double layer. The middle graph panel shows a representative × profile recorded on the sample surface. Positions of the height maxima are marked by diamonds symbols. The full symbols belong to one layer and open to the second one with the same periodicity of 0.32 mm and shift 0.13 mm. The lower panel shows an example of Y profile recorded on the ridge of a “hilled row”. The total thickness of the printed motif varies between 1 and 3 µm.

**Figure 8:**
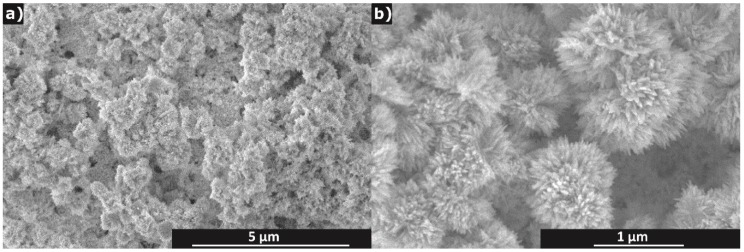
SEM images of printed CuO sensitive layer on Ag layers at low (**a**) and high (**b**) magnification.

A sensitive CuO layer was printed on top of the Ag pattern, as can be seen in Figure S4 in the Supplemental Information. It is documented by SEM images in Figure 8 that the CuO layer is relatively compact and the particles kept their original morphology. The flowerlike particles were not either damaged, lost petals or modified in any other way by the processes of ink preparation and passage through the printing nozzle.

The sensing layer was printed five times in order to improve the coverage, thickness and homogeneity of the CuO layer. Trapping of layers reduces the roughness of the final relatively to the total thickness of the printed multilayer. The AFM measurement confirmed the enormous roughness at the nanoscale expected according to the observation of synthesized nanostructured CuO microparticles. Typical AFM images of surface topography at the nanoscale for a single layer and for five layers on each other are shown in Figure S5 in the Supplemental Information. It is obvious that there is no difference between samples in terms of maximum and minimum height span, as it was always the last printed layer which was examined and that surface of only several adjacent particles was scanned. On the other hand, average numbers show a difference. The roughness of a single CuO layer is 2770 ± 140 nm and of five CuO layers the roughness is 170 ± 70 nm.

**Figure 9:**
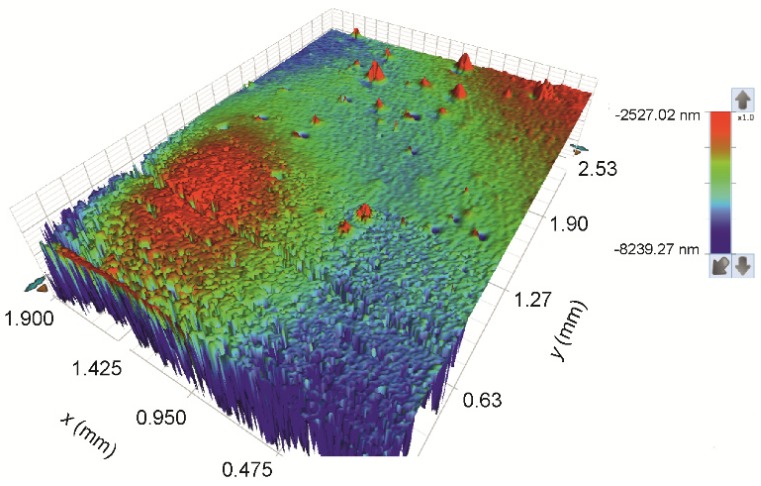
3D topography of printed CuO layers.

The CuO ink (Ink 4) has a much smaller content of the solid phase than the silver dispersion. This is another reason for finishing of the device by four overlays printed on top of the prime CuO layer in order to obtain a homogeneous sensing layer. Numerous overprints of the substrate smeared the ridged morphology of the surface of silver electrodes and a relatively smooth surface envelope reproduced the basic shape of the interdigitated electrodes. However, the surface is still very rough at the small scale due to the enormous CuO layer porosity and morphology of the CuO particles. In fact, this is a suitable morphology for a VOC sensing device. Figure 9 gives an overview of the 3D topography of the final surface of the printed sensing device.

### 3.2. Results and Discussion of the Electrical Characterization and Sensing Properties

#### 3.2.1. Preliminary Considerations and Experimental Verification of Printed Material Layers, Electrodes and Wirings

Prior fabrication of devices, thin continuous square shaped 2 cm × 2 cm films deposited on the original substrate were prepared. Single layer material samples were printed with the aim to characterize the conductivity of basic material components of the multilayer device. The resistivity of a printed silver single layer was (2.4 ± 0.6) × 10^−7^ Ω∙cm which is less than one order of magnitude only far from the well-known standard value for bulk silver metal (6.3 × 10^7^ S/m or 1.59 × 10^−6^ Ω∙cm), which can be considered sufficient result for a single layer printout with relatively porous morphology as discussed above. On the other hand, the resistivity of the single CuO layer was immeasurably high and exceeded the limit >10^4^ Ω∙cm of the four-point probe apparatus designed for measurement of conductive samples. Therefore, only one overlay was chosen for silver and four overlays were chosen for CuO layer printing in the fabrication of testing devices.

The sensor response and its recovery were investigated by repeated exposure to vapours of ethanol, methanol and water as representative examples of targeted gas analytes at a temperature of 25 °C. Firstly, the response of bare interdigitate silver electrode structures was tested. No exposure to any of the tested conditions delivered a measurable resistance response. In all cases, overload was signaled by the measuring apparatus which indicated that the resistance of the incomplete device was higher than 40 MΩ, which is the maximum range of the used multimeter. Any contribution of the surface of the flexible PET substrate to the response of fabricated devices is excluded. Similarly, the suitability of five times-printed CuO film was preliminarily confirmed.

#### 3.2.2. Humidity Sensing and Why Its Mechanism Differs from the Common p-Type one for CuO

**Figure 10:**
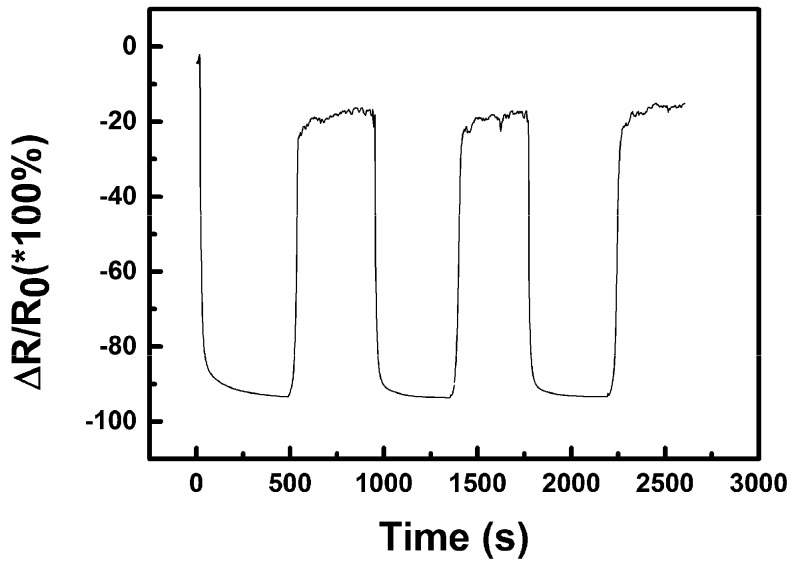
Relative resistance change (Δ*R*/*R*_0_) of the nano-copper oxide sensor within water sensing at 25 °C (*R*—actual resistance value, *R*_0_—initial resistance; *R*_0_ = 40 MΩ).

Figure 10 shows typical resistance change of the device prepared with the best ink (Ink 4) composition within the saturated water vapour sensing test at 25 °C. Both on- and off-values of resistance show relatively fast saturation and virtually approach a constant value after several minutes and the sensor has good reversibility of the signal over several tested complete cycles of the sensor testing procedure. However, the recovery of the resistance of the sensor was incomplete due to the relative humidity of the air in the laboratory. The sensor was actually cycled between ambient humidity in the laboratory where RH was 45% (off state) and in saturated vapours with RH = 100% (on state). Since the experiment took typically less than one hour, the air humidity in the laboratory was stable enough to cause no baseline drift. It can be said that both the saturation of the sensor in vapours and recovery outside the vapour environment have approximately the same rate. Such behaviour indicates the reversibility of the sensing process with a steady reference off-state value of the resistance, although the use of a vapour-gas mixing unit and a controlled testing chamber will be necessary to more thoroughly perform studies in further research.

The sensing principle of CuO-based devices has been numerously reported for high temperature-operated sensors [16,31,32]. The sensing mechanism of gas sensors made from p-type semiconductors such as CuO is different from the much more frequently utilized and known n-type metal oxides. In air, oxygen molecules adsorbed onto the surface of CuO trap electrons and, thus, form oxygen ions which causes bending of the energy band upwards at the surface [33]. This results in an accumulation of positively-charged holes at the CuO particle surface because the trapped electrons leave holes according to the following mechanism [26,33,34]:

(1) $$O_{2}\;↔\;O_{2}^{-}\;+\;1hole^{+}$$

(2) 12 O2 ↔ O2− + VacancyCu2++ 2hole+

Adsorbed molecular oxygen (O_2_) can be ionized preferentially to molecular O_2_^−^ at temperatures below 200 °C and to monoatomic ions O^−^ and O^2−^ predominantly at temperatures higher than 200 °C.

When the surface is exposed to water vapour, the double ionized oxygen (Equation (2)) reacts with H^+^ ions coming from the dissociation of water vapour to form OH^−^ as the equation below [35]:

(3) $$H^{+}+O_{2}^{-}\;↔\;OH^{-}$$

This reaction leads to a release of the trapped electrons and neutralization of the holes in p-type CuO. Moreover, it can be generalized to any reducing species (such as NH_3_ or alcohols, etc.) that can be oxidized and release a negative charge (electron). The decrease in the concentration of holes results in an increase of the resistance in the surface layer of CuO. Meanwhile, the number of oxygen ions O^−^, O^2−^ and O_2_^2−^ absorbed on the surface of CuO is reduced as well, which leads to the decrease in the magnitude of the negative quasi-gate which results in further decrease in conductivity [6,16]. Therefore, a monotonous increase of the resistance with the increase of relative humidity is experienced if the CuO sensor is operated commonly at high temperatures.

Nevertheless, it is clearly documented in Figure 10 that the resistance of our sensor decreases with an increase of humidity, which is the opposite of previously described behaviour. It has been already published that the response of the sensor is largely affected by the device operation temperature, mainly due to the temperature dependence of adsorption-desorption kinetics, as well as reaction rates and equilibriums occurring on the sensing material surface [22,36]. Ceramic and semiconductor sensors are usually operated at higher temperatures of several hundreds of °C which assures surface cleanliness, while precise maintaining of a constant temperature provides the sensor with response stability. Fully-printed sensors on common plastic (PET polymer) substrate cannot be either prepared or operated at such high temperatures and the above described microphysical mechanism can be hardly the main one in action. At the laboratory temperature, molecular forms of adsorbed oxygen prevail over atomic ions as well the equilibrium in Equation (1) should be shifted to the left side of the reaction, which means that reactants are strongly favoured over the ionic product. Moreover, the sensing layer consists not only of pure CuO nanoparticles able to interact with gaseous species, but contains surfactant and the dispersing agent in non-negligible amounts. The primary physical p-type semiconductor surface reaction principle is most likely not active and replaced or outweighed by other mechanism(s) in such a complex system.

To analyse the other possible mechanisms, morphology the active layer shall be considered first. As shown by SEM, AFM and profilometric study, the layer has a sponge-like morphology with the pore diameter varying from nano- to micrometers. The morphology of the porous structure is developed on two levels. To begin with single particles, it must be reminded that each chrysanthemum-like nanostructured particle has its internal porous structure as shown in Figure 3. The anchoring of CuO nanosheets to a common centre in the flower creates large free space between individual petals. Moreover, this expanded configuration of petals is stable and avoids collapse of the voluminous structure due to the stacking of nanosheets which would occur if exfoliated nanosheets are used because they can spontaneously assemble during drying of the ink dispersion. Preservation of the flower-like assemblies in finished layers is documented in Figure 8. Higher hierarchical level of porous morphology is created by deposition of the ink and assembling of nanostructured CuO “flowers” into a thin film by its drying. The contiguity of single printed layer is too low, which results into practically immeasurable resistance (see Section 3.2.1 and the first paragraph of this section), therefore, four additional layers needed to be trapped on the primary one to obtain an applicable film which was manifested by a significant decrease of its roughness. Resistance of such a printed motif was then suitable over the tested range of parameters. Humidity cannot only be adsorbed on the solid surface, but may capillary condense in the microporous structure and dissolve the residual additives. A report on porous systems with high porosity and, hence, possibly poor contact between particles and operated at room temperature [21], described a similar behaviour to our system. The otherwise very authoritative review [6] left the occurrence of such behaviour uncommented. According to the review [37], the conductivity is increased by moisture condensation inside the pores due to both electron transfer and proton transfer mechanisms and possibly by the increase of ionic conductivity if some soluble ionic species are available in the material. Since the portion of solid residuals from the additives in the ink formulation should be relatively high, their effect on sensor sensitivity and sensing behaviour cannot be neglected. The surfactant (BYK^®^ 348) is a non-ionic compound polyether-modified siloxane which is not expected to contribute directly to the conduction mechanism. Formation of hydrogen bonds with the oxygen atoms in this polymer would most likely impede the proton transfer mechanism. On the other hand, the polymeric part of the dispersant (DISPERBYK^®^ 190) is a high molecular weight block co-polymer with carboxyl groups that have high affinity to the surface of the nanostructured CuO particles. The adsorbed ionic groups on the semiconductor surface can affect the surface electron transport (tunnelling effect), moreover, the proton transfer mechanism may be influenced by hydrolytic equilibrium as well as by the involvement of carboxylic groups in hydrogen body formation, as well. It can be expected that some carboxylic groups in the DISPERBYK co-polymer are neutralised and that the polyelectrolyte-based humidity response contributes to the sensing mechanism as well. According to our opinion, it is reasonable to expect that it is not only moisture which can influence the sensing layer properties by capillary condensation. Any solvent that is miscible with the original ink composition, like alcohols, which we tested too, can contribute to the conductivity increase if condensed inside the porous structure due to interactions with chemisorbed and physisorbed layers of water.

In addition, the method of the sensing device resistance measurement itself may have impact on the measured result. An affordable and simple digital multimeter is often used for efficient measurements of higher values of resistance, similarly to our case. Such a practical apparatus employs a two-wire method that is suitable for measuring values above 100 Ω up to several tens of MΩ, when high accuracy is not required. The principle is as follows: test current is forced through the measured device and the multimeter measures voltage at its terminals. The test current is commonly measured as a voltage drop on an internal standard resistor of known resistance inside the multimeter. Change of the internal resistor can be used for choice of resistance measuring ranges on the multimeter. Other specific variations of the internal measurement setup can be employed, but the general principle of two-wire measurement is used in all apparatuses of this kind. An important fact is that the applied voltage changes with the resistance of the measured sample (device) which may result in nonlinearity of its response if the sensing device is not of purely ohmic character.

**Figure 11:**
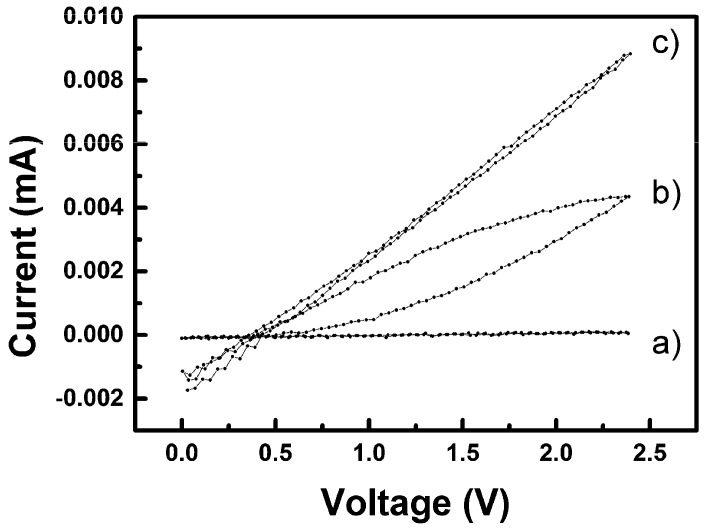
I-V characteristics of CuO sensors measured at 25 °C in water vapours, (**a**) outside from vapours, (**b**) hysteresis, and (**c**) stabilized.

In order to investigate all above mentioned issues, the current-voltage (I-V) characteristics of the sensor were recorded. Figure 11 show I-V plots of a specimen measured at 25 °C under three different stages of the testing cycle. It took 60 s to record each curve (i.e., from 0 V up to 2.5 V and back down to 0 V). Curve a) was recorded for the sensor stabilized in the air atmosphere. As can be expected, the resistance of the device is high (calculated from the slope ca. 13 MΩ, keeping in mind that the sensor was not preconditioned in a desiccator but stabilized in the ambient atmosphere) and there is no hysteresis observed. The second curve b) was recorded for the device after insertion into the saturated vapour, i.e., before signal saturation. The change of the resistance value is manifested in a well-developed hysteresis, which shows a continuous decrease of the resistance of the device. The last curve c) was recorded for the device equilibrated for five minutes in the saturated vapours. Resistance of the device is obviously stabilized at a constant value and is about 0.2 MΩ (calculated from the slope). The cross-section of the I-V curve with the x axis was always at about 0.35 V. It must be reminded that the used electrical source was asymmetric (actually not grounded, hence, floating) and the I-V characteristics were the same regardless to the polarity of the device connection. Indeed, the device is of symmetric design, however, the (0.35 ± 0.02) V offset always developed in the same manner and vanished after the device was taken out from the vapour environment. The value 0.34 V is the standard reduction potential at 25 °C for the following reaction:

(4) $$Cu^{2+}\left(aq\right)+2e^{-}\;\to \;\;Cu^{0}\left(s\right)$$

As a reasonable explanation it can be suggested that an electrochemical cell was temporarily developed each time when the device was exposed to higher voltage due to reduction of a small amount of copper from CuO in the vapour environment which created a liquid electrolyte environment by condensation in the pores of the sensing layer, thus enabling the redox reaction. If created, the Cu phase must be highly dispersed with a large specific surface and, therefore, oxidized immediately after disconnection and removal from the vapour environment due to oxygen in the air. Thus, we observed the same behaviour in all tests taking the device into the testing chamber and out, changing wire connections’ polarity from time to time. This may have implications towards linearity of the sensor’s response in some specific electrical connections (if higher voltage than the reduction potential is used for resistance measurement).

#### 3.2.3. Sensing of Alcohol Vapours and Re-Discussion of the Sensing Mechanism

Similar sets of data were recorded for ethanol and methanol. Additionally, in this case, each experiment took less than one hour, so the air humidity in the laboratory was stable enough to cause no baseline drift and the observed resistance changes can be ascribed solely to the sensing of alcohol vapours, although the general level of the sensor’s response shall be influenced by the humidity as well. Further studies will be necessary to analyse the simultaneous effect of moisture and alcohol vapours on the response of the sensor, however, it can be already said that a low cross-sensitivity can be expected.

**Figure 12:**
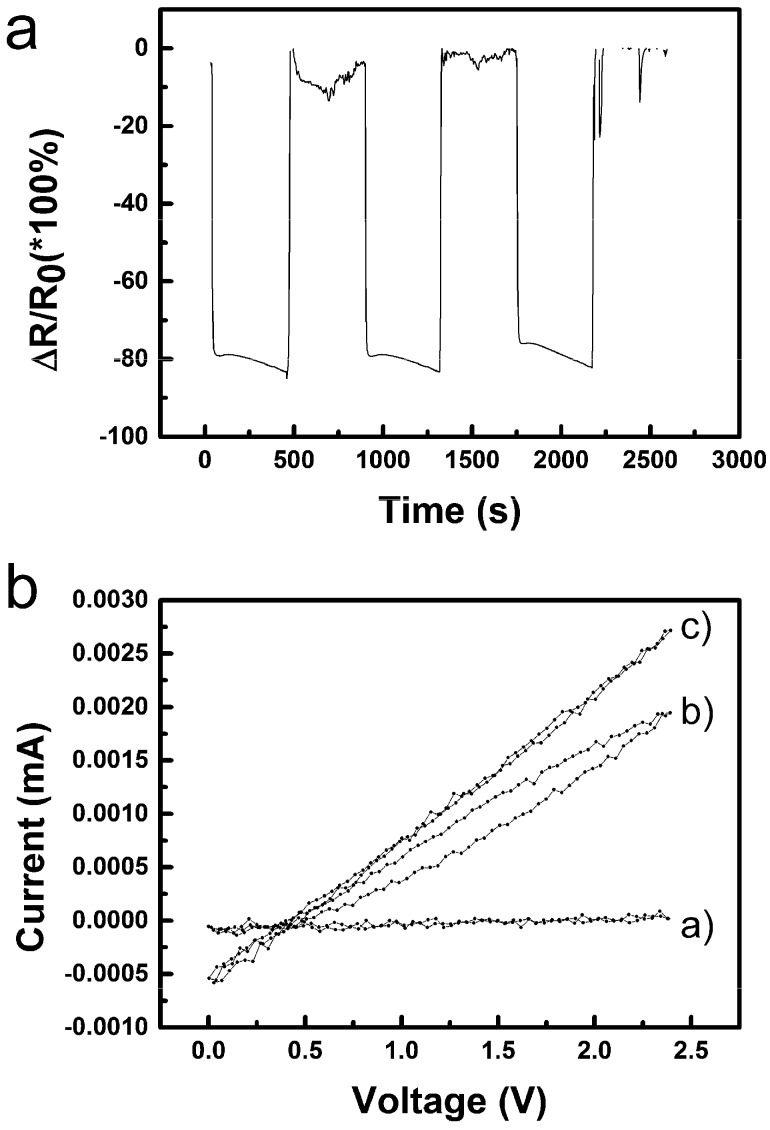
(**a**) Relative resistance change (Δ*R*/*R*_0_) of the nano-copper oxide sensor within ethanol sensing at 25 °C (*R*—actual resistance value, *R*_0_—initial resistance; *R*_0_ = 40 MΩ); (**b**) I-V characteristics of CuO sensors measured at 25 °C in ethanol vapours, (**a**) outside from vapours, (**b**) hysteresis, and (**c**) stabilized. The missing values in the upper graph (**a**) are due to overflow of the measuring range of the instrument.

The record of three cycles for ethanol (see Figure 12a) showed roughly similar behaviour to water, but both the saturation of the sensor after insertion into vapours and its recovery after being removed is much faster than in the case of water. Moreover, the recovery to initial value reaches the resistance limit for the used multimeter as seen by cut data at the 0% level. Such situations can make the use of the sensor impracticable. A more detailed inspection of the graph in Figure 12a revealed that the saturation of the signal is reached quite quickly, possibly due to easier adsorption and/or diffusion of ethanol into the active layer. However, the saturation is quickly outweighed by another process which results in a continuous steady (linear) decrease of resistance unlike a simple exponential saturation observed for water. Both observations can be explained by relatively slow replacement of adsorbed moisture in the sensing CuO layer by alcohol molecules since partial pressure of water in saturated vapours over absolute ethanol is certainly much lower than in the ambient air in the laboratory. Measured I-V characteristics for ethanol shown in Figure 12b are again roughly similar to those recorded for water. Three characteristic curves (a) for sensors outside vapours, (b) with hysteresis, and (c) with a slope of approx. 0.7 MΩ at a more or less equilibrated state were recorded. Interestingly, the voltage offset is shifted slightly to higher values up to 0.42 ± 0.05 V. It can be hypothesised that, besides copper reduction, oxidation of ethanol to acetaldehyde can be a simultaneous process which can also contribute to the observed linear decrease of the sensor’s resistivity when exposed to vapours of ethanol.

**Figure 13:**
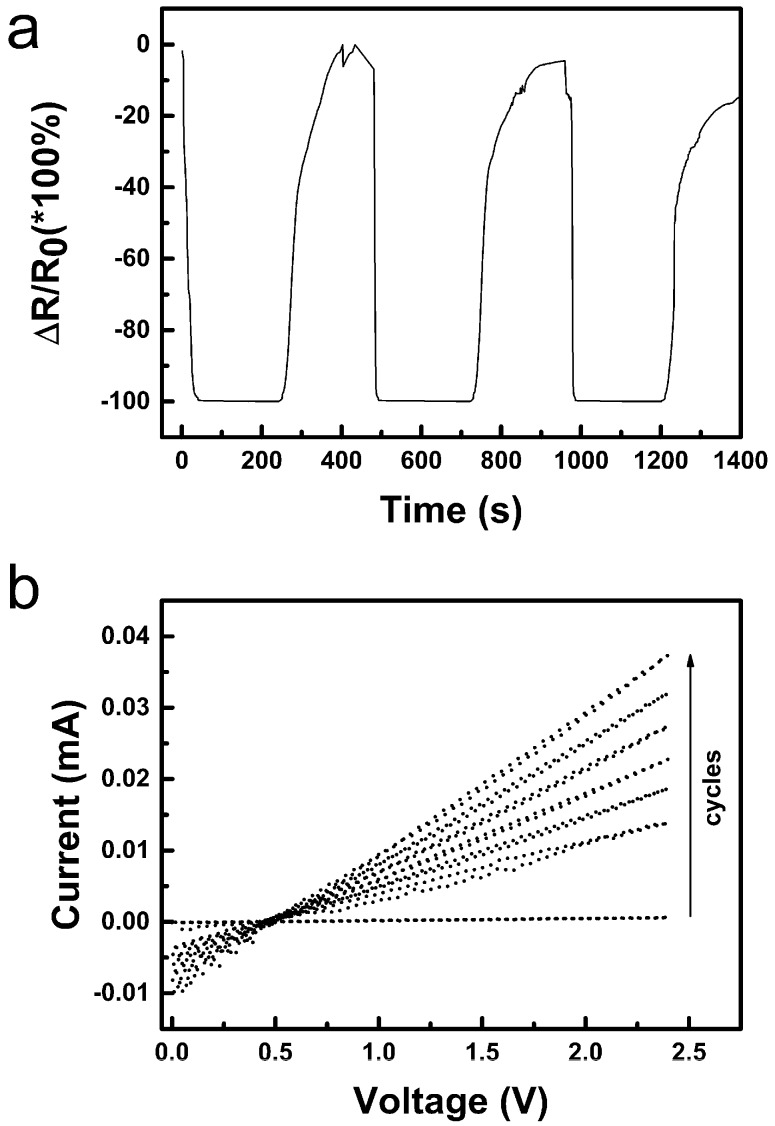
(**a**) Relative resistance change (Δ*R*/*R*_0_) of the nano-copper oxide sensor within methanol sensing at 25 °C (*R*—actual resistance value, *R*_0_—initial resistance; *R*_0_ = 40 MΩ); (**b**) I-V characteristics of CuO sensors measured at 25 °C in methanol vapour. The arrow shows the sequence of the cycles. The I-V characteristics were recorded in a sequence without any delay during the first exposure of the sensor to methanol vapour.

Methanol was another investigated organic volatile liquid. It can be seen on the three cycles in Figure 13a recorded for the sensor insertion into and taking out of saturated vapours of methanol that the Relative resistance change reaches nearly down to −100%, which corresponds to the decrease of resistance to several tens of kΩ. While the resistance decrease after insertion of the sensor into methanol vapours seems to be much faster than in case of water, the recovery of the sensor is significantly slower and even slowed more at −40% which may indicate that two processes are manifested in the recorded curve.

A record of I-V characteristics for the sensor in methanol vapours is shown in Figure 13b. The measurement was started before insertion of the sensor into vapours environment and then continued cycle by cycle up to nearly saturated state with the slope approx. 0.05 MΩ. In total the record took seven minutes. According to that, it can be said that the resistance measurement did not show the saturation kinetics optimally because of the linear (percentage) scale and that there is a continual decrease to lower resistances similarly as in the case of ethanol but the process is either of much larger extent or much faster. It might be, that some product of this processes are generated and accumulated in the sensor and its release is manifested during the sensor recovery on the dry air atmosphere also. A notable characteristic is the voltage offset of 0.46 ± 0.03 V again. The potential necessary for simple oxidation of a primary alcohol to the corresponding aldehyde (formaldehyde in case of methanol) might be estimated to be +0.1–0.2 V, which coincides with the increase of the voltage offset in comparison with that observed for moisture exposure. It can be expected, that methanol has high affinity to the dried materials present in the active sensing layer similarly to ethanol, but it has higher diffusion coefficient and eventual reactivity. It should be also mentioned, that methanol is the most volatile among tested compounds and has the highest absolute partial pressure of its saturated vapours among already tested liquids (i.e., higher than ethanol, which, again, has higher partial pressure than water vapour). Based on the presented data only it cannot be distinguished which processes exactly took place in the sensor, however, it can be said that the sensing mechanism is not the one typical for p-type semiconductors at higher temperatures, but most likely it is primarily based on adsorption/desorption and condensation of the volatile compounds in the active layer accomplished by subsequent electrochemical reactions induced by voltage applied on the electrodes of the device in order to measure its resistance.

## 4. Conclusions

A series of inkjet inks was prepared from copper(II) oxide nanostructured particles synthesized by MW enhanced solvothermal technique. The particles were dispersed in water with the aid of two surface active compounds. It was found that the addition of a mixture of the dispersant and the stabilizer affects the viscosity of inks more than the content of CuO itself. On the other hand, the surface tension of all tested dispersions was reduced sufficiently to enable befitting compatibility with the substrate as well as with pre-printed silver interdigitated electrodes. A good printability was achieved by the optimization of the printing process parameters with the aid of a dimensionless criteria printability evaluation model and the best performing dispersion (Ink 4) was used for the fabrication of CuO sensing devices.

The application potential of nanostructured copper(II) oxide films on flexible substrate for the methanol, ethanol, and humidity sensing was demonstrated at the operating temperature of 25 °C. Good sensitivity and response reversibility of the CuO film-based sensor was experienced although no significant selectivity to the used alcohols and water was observed. In addition to the morphology of individual particles, the porous structure of active layer and the presence of both the surfactant and the dispersant, which remain in the sensing CuO layer, affect its final properties. The sensing mechanism is different from the one typical for a p-type semiconductor-based sensor operated at high temperatures. At laboratory temperature, it is most likely a complex mechanism, which involves adsorption/desorption and capillary condensation of moisture or alcohol vapours in the pores of the active layer followed by electrochemical reactions driven by voltage applied to the device to measure its resistance response. An offset about 0.35 V in I-V characteristics recorded for devices exposed to saturated vapours points towards an electrochemical reaction which might be the reduction of CuO to Cu^0^ in the sensing mechanism. Oxidation of primary alcohols to corresponding aldehydes can be hypothesised as an additional plausible process that influences the sensing mechanism of alcohols, too. Regardless to what is the possible source of sensor’s response nonlinearity, more attention should be paid to the method of resistance measurement itself. Definition of an operating point and keeping the voltage applied to the sensor constant may be more suitable than to rely on use of ohmmeters.

## 5. Patent

The work on inks reported in this article partially resulted into the Czech patent ‘Inorganic ink based on nanoparticles, especially for material printing’, no. 307435; 2018.

## Figures and Tables

**Table 1:** Composition, viscosity, surface tension and density of CuO ink and solvent at 25 °C. Note that the dispersant contains only 40 wt% of non-volatile co-polymer.

Ink	Surfactant (wt%)	Dispersant (wt%)	CuO (wt%)	Viscosity (mPa∙s)	Surface Tension (mNm^−1^)	Density (g∙cm^−3^)
**Ink 1**	21.60	7.20	3.40	(4.66 ± 0.04)	(22.13 ± 0.09)	(1.04 ± 0.04)
**Ink 2**	14.20	14.20	4.80	(7.13 ± 0.02)	(21.98 ± 0.04)	(1.05 ± 0.06)
**Ink 3**	13.90	13.90	6.60	(6.53 ± 0.01)	(21.73 ± 0.08)	(1.04 ± 0.03)
**Ink 4**	7.20	21.60	3.40	(6.59 ± 0.02)	(21.42 ± 0.08)	(1.04 ± 0.07)
**Ink 5**	12.88	19.32	7.20	(10.54 ± 0.01)	(21.52 ± 0.05)	(1.09 ± 0.04)
Water	-	-	-	(1.00 ± 0.02)	(72.22 ± 0.08)	(0.99 ± 0.01)
CuO + water	-	-	3.00	(1.18 ± 0.02)	(71.56 ± 0.01)	(1.00 ± 0.02)

**Table 2:** Values of selected dimensionless criteria for CuO inks, dispersion of CuO in water and water alone at 25 °C based on data from the previous table using a velocity of 4 m/s and characteristic length of 21.5 µm. For the formulas, see Appendix A.

Ink	Dimensionless Criterion				
	*Re*	*We*	*Oh*	*Z*	*Ca*
**Ink 1**	18.7	15.8	0.212	4.72	0.84
**Ink 2**	12.4	16.1	0.324	3.09	1.30
**Ink 3**	13.4	16.1	0.300	3.34	1.20
**Ink 4**	13.3	16.3	0.305	3.28	1.23
**Ink 5**	8.7	17.0	0.475	2.11	1.96
Water	83.2	4.6	0.026	38.75	0.06
CuO + water	71.2	4.7	0.030	32.85	0.07

## Supplementary Materials

The following is available online at https://www.mdpi.com/1424-8220/19/14/3068/s1, Supplementary information file in pdf format which contains Figure S1: Image of droplets formation and ejection—left part, waveform—right part, Figure S2: A micrograph of a part of Ag interdigit surface, Figure S3: Profile analysis of printed Ag layers, Figure S4: A micrograph of a part of Ag interdigit with CuO layer on top, Figure S5: Representative 3D views of peak force mode AFM images of a single CuO layer (1) and four layers on each other (2).

## Appendix A. Used Dimensionless Criteria

Following dimensionless correlations were used in the article:

(A1) $$\begin{array}{cc} \mathrm{Reynolds}\;\mathrm{number}: & Re=\frac{\upsilon \cdot \rho \cdot A}{\eta } \end{array}$$

(A2) $$\begin{array}{cc} \mathrm{Weber}\;\mathrm{number}: & We=\frac{v^{2}\cdot \rho \cdot A}{\sigma } \end{array}$$

(A3) $$\begin{array}{cc} \mathrm{Ohnesorge}\;\mathrm{number}: & Oh=\frac{\sqrt{We}}{Re}=\frac{\eta }{\sqrt{\sigma \cdot \rho \cdot A}}=Z^{-1} \end{array}$$

(A4) $$\begin{array}{cc} \mathrm{Capillary}\;\mathrm{number}: & Ca=\frac{We}{Re}=\frac{v\cdot \eta }{\sigma } \end{array}$$

Physical quantities (material, process and tool parameters) used in these formulas are as follows: ρ is the density of the fluid, υ is the velocity of the fluid, η is the dynamic viscosity of the fluid, σ is the surface tension of the fluid and *A* is a the characteristic length (the hydrodynamic equivalent diameter of the printing nozzle). The *Z* number is calculated as the inverse of the Ohnesorge number.
