# Supplementary material for: Fully Inkjet-Printed CuO Sensor on Flexible Polymer Substrate for Alcohol Vapours and Humidity Sensing at Room Temperature
Source: Sensors (Basel). 2019 Jul 11;19(14):3068. doi: 10.3390/s19143068 (PMC6678509; doi:10.3390/s19143068)
Supplement: Supplementary file 1 [file sensors-19-03068-s001.pdf]

sensors

MDPI

Supplementary information

Fully inkjet-printed alcohol vapours and humidity CuO sensor on flexible polymer substrate for operation at room temperature

Petr Krčmář, Ivo Kuřitka*, Jan Mašlík, Pavel Urbánek, Pavel Bažant, Michal Machovský, Pavol Šuly, Petr Měrka

Centre of Polymer Systems, University Institute, Tomas Bata University in Zlin, Trida Tomase Bati 5678, 760 01 Zlin, Czech Republic

* corresponding author: ivo@kuritka.net

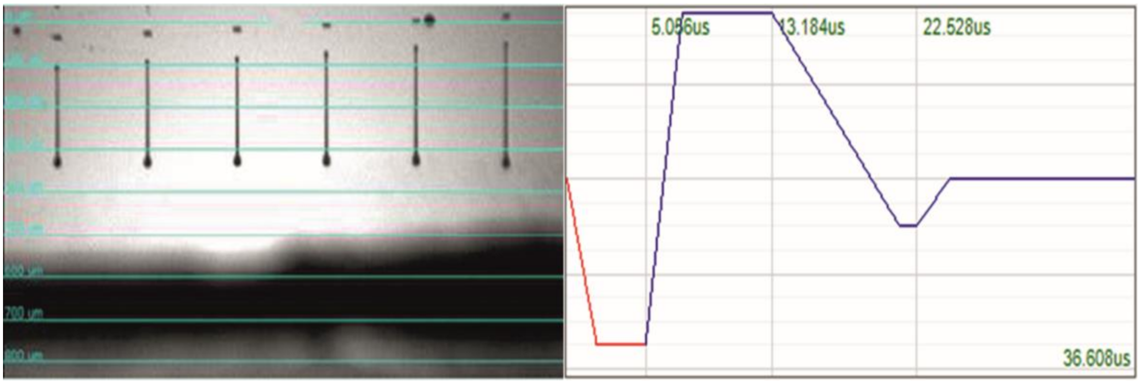

Figure S1. Image of droplets formation and ejection – left part, waveform – right part, used CuO concentration 3.5 Wt.% in water/DMSO mixture, firing voltage 24 V approximately, temperature 30 °C.

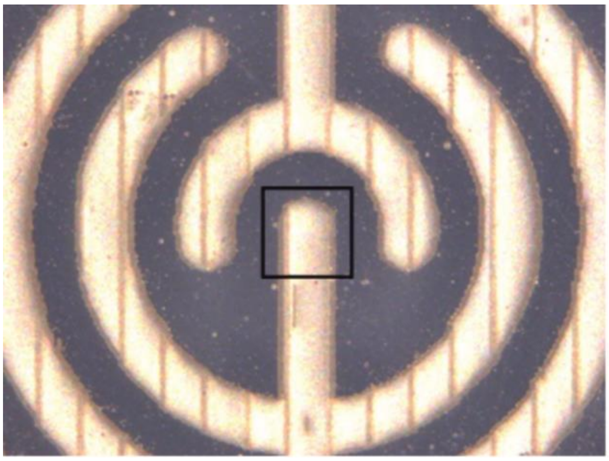

Figure S2. A micrograph of a part of Ag interdigit surface. The black line rectangle in the middle marks area of the interdigit surface used for further SEM analysis. The length of its side is 0.8 mm. Image captured by the optical microscope.

sensors

MDPI

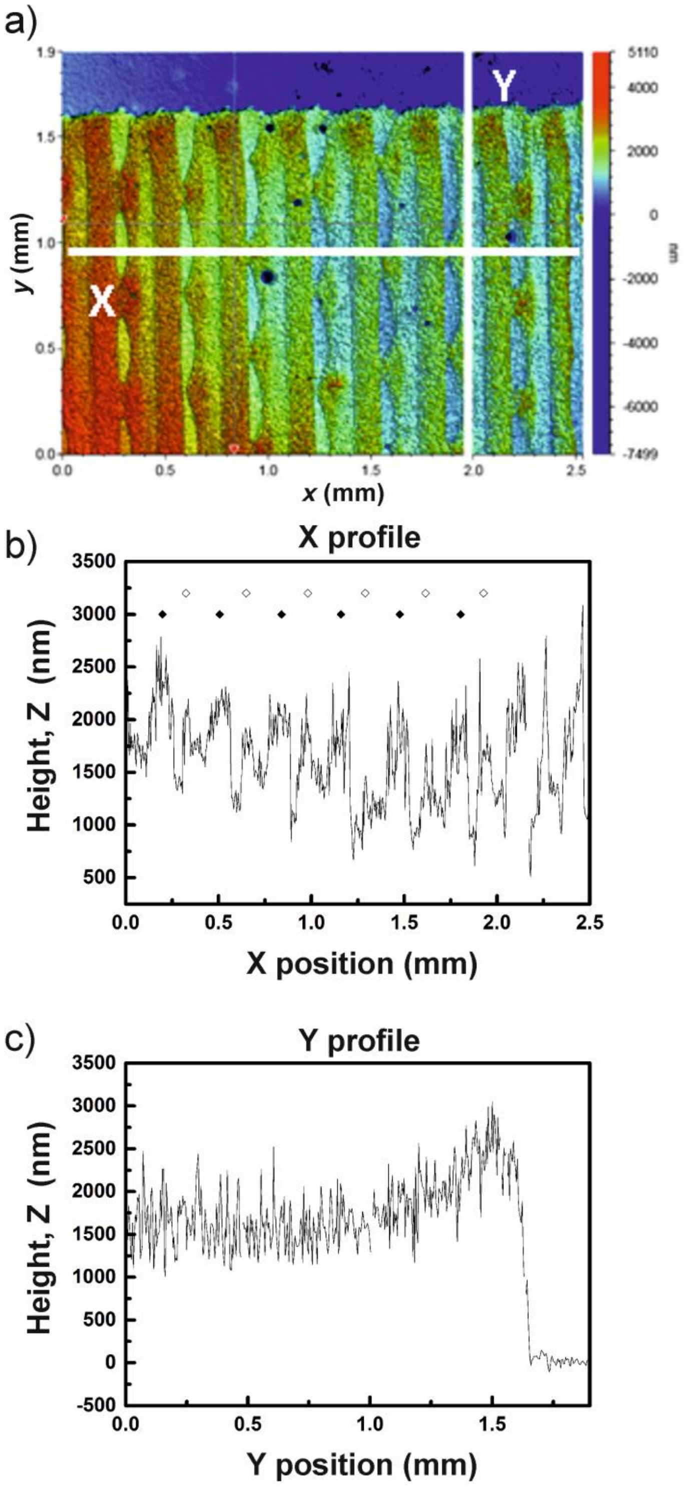

Figure S3. Profile analysis of printed Ag layers: a) Top view of printed Ag layers, the same area is shown as in figure 10; b) X profile – for symbol explanation see text; c) Y profile

sensors

MDPI

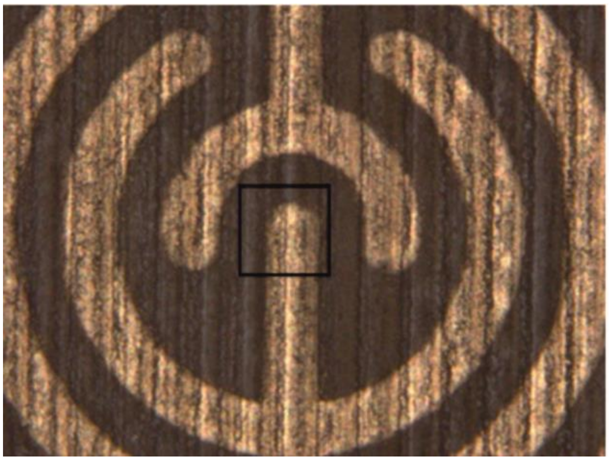

Figure S4. A micrograph of a part of Ag interdigit with CuO layer on top. The black line rectangle in the middle shows area for SEM analysis. The length of its side is 0.8 mm. Image was captured by optical microscopy.

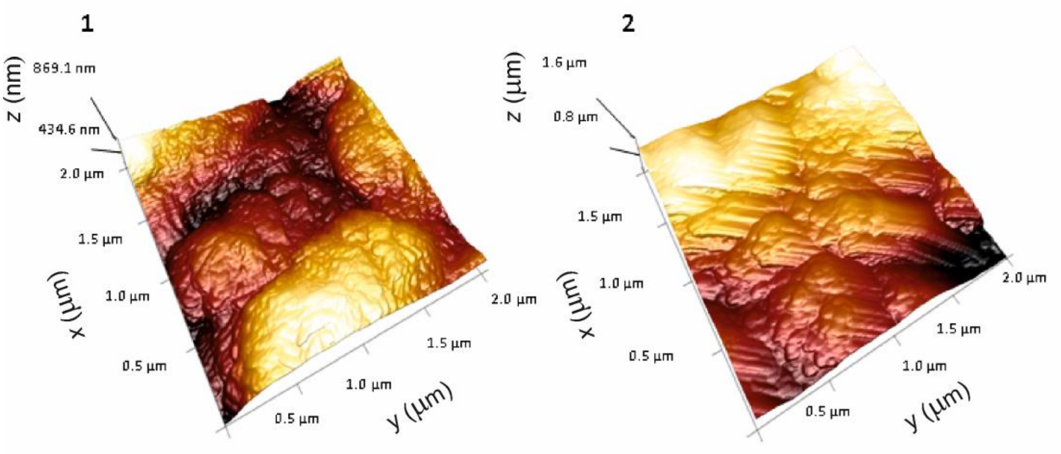

Figure S5. Representative 3D views of peak force mode AFM images of a single CuO layer (1) and four layers on each other (2). Scan area size was 2x2 µm.
